# Design and development of pH-sensitive nanocarriers using molecularly imprinted polymers for the targeted delivery of sodium thiopental†

**DOI:** 10.1039/d4na00926f

**Published:** 2025-02-18

**Authors:** Ayda Yari-Ilkhchi, Abdolrahim Abbaszad Rafi, Mehrdad Mahkam

**Affiliations:** a Faculty of Science, Chemistry Department, Azarbaijan Shahid Madani University Tabriz Iran mmahkam@yahoo.com mmahkam@gmail.com mahkam@azaruniv.ac.ir ayda.yari90@gmail.com; b Faculty of Chemical and Metallurgical Engineering, Department of Chemical Engineering, Istanbul Technical University Maslak 34469 Istanbul Turkiye; c Faculty of Engineering and Natural Sciences, Sabanci University 34956 Istanbul Turkiye; d FSCN Research Center, Mid Sweden University Holmgatan 10 851 70 Sundsvall Sweden

## Abstract

Sodium thiopental (STL) is an ultrashort-acting barbiturate that acts quickly on the brain, reduces levels of adrenaline, noradrenaline, and dopamine, and has neuroprotective properties. However, its side effects, especially in high doses, can be severe, including respiratory failure and cardiac complications. Molecularly imprinted polymers (MIPs) are three-dimensional polymeric networks that mimic the structure and functionality of target molecules. MIPs include benefits such as stability, selectivity, and cost-effectiveness. Combination with magnetic nanoparticles (MNPs) not only enhances their stability and biocompatibility but also provides magnetic separation capabilities. This research introduces the design and synthesis of pH-sensitive MIPs as targeted nanocarriers for the selective uptake and controlled release of STL molecules. The MIPs were synthesized in various forms, including magnetic core MIPs (MMIPs), standard MIPs (MIPs), and fiber-shaped MIPs (MIP_F_), to explore their comparative efficiency and structural advantages. Bemegride (BMG), an antidote structurally similar to STL, was utilized to evaluate the selectivity of these MIP systems. The formation of specific binding sites of STL on MIPs during the polymerization process leads to selective recognition and matches STL's shape, size, and functional groups. In this regard, all types of MIPs exhibited significant rebinding affinities over their non-imprinted polymer (NIP); specifically, MMIPs displayed a high affinity for uptake of STL (393.8 ± 1.328%) against BMG (360.72 ± 6.72%) over 24 h. The pH sensitivity of the nanocarriers was investigated in simulated gastric fluid (SGF) and simulated intestinal fluids (SIF) environments. The quantitative results indicated that the prepared nanocarriers showed a controlled release in SIF environments. MMIPs achieved a release efficiency for STL and BMG of approximately 57.7 ± 0.6% and 85.4 ± 4.6%, respectively, over a 78-hour period. These findings highlight the potential of MMIPs for dual-uptake and targeted release applications of STL in specific pH environments.

## Abstract 2

This research introduces the design and synthesis of pH-sensitive MIPs, including magnetic, nonmagnetic, and fiber, as targeted nanocarriers for the selective uptake and controlled release of STL molecules in SGF and SIF environments.

## Graphical abstract

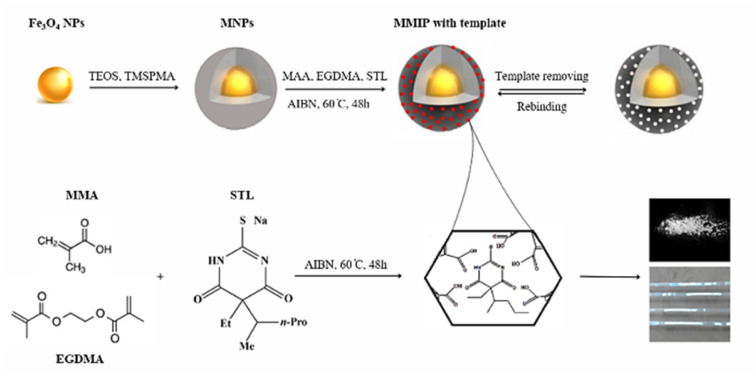

## 1. Introduction

Barbiturates and their numerous derivatives have been produced and utilized in medical applications as anxiolytics, sedatives, anesthetics, and antiepileptics.^1^ These compounds exert their effects on ion channels, primarily by enhancing the inhibitory activity of Cys-loop receptors (GABA_A_ and nACh). At higher concentrations, barbiturates may also inhibit excitatory glutamate receptors, including α-amino-3-hydroxy-5-methyl-4-isoxazolepropionic acid (AMPA) and kainate receptors.^2,3^ STL is categorized as an ultrashort-acting barbiturate^4,5^ that is immediately taken up by the brain^6^ and decreases the quantity of endogenous adrenaline, noradrenaline, and dopamine in animals.^7^ In addition, it exhibits bronchospasm *via* inhibiting sympathetic activity and has neuroprotective properties against cerebral ischemia.^8,9^ If taken alone, the adverse effects may restrict its utility. High doses of STL can result in poisoning and mortality by respiratory failure, cardiac depression, and cerebral vascular complications.^10^ In order to overcome poisoning and intoxication, BMG was used as an antidote.^11^ BMG stimulates the respiratory and central neurological systems, therefore counteracting STL's depressive effects. It aids in the restoration of normal respiration and awareness in patients who have received high amounts of thiopental and experienced significant sedation or overdose.^12,13^ Therefore, an ideal anesthetic agent should have easy and quick induction, fast recovery, intraoperative amnesia and analgesia, and no postoperative adverse effects.^14^

MIPs are utilized as artificial recognition systems in a variety of scientific applications, including solid-phase microextraction,^15–17^ catalysis,^18^ drug delivery,^19^ artificial enzyme inhibitors or antibodies,^20^ liquid chromatography,^21^ and electrochromatography.^22^ In considering this, MIPs are being employed in drug delivery systems to address drug overdoses and side effects, transport them to a specific location, and control their pharmacological responses.^23,24^

The MIP approach is based on a polymerization reaction that involves the combination of a functional monomer(s), template molecules, crosslinker(s), and initiators in an appropriate solvent.^25^ A three-dimensional polymer network can result from either non-covalent or covalent interactions.^26^ Purification and template removal are critical procedures in the process of creating binding sites that are similar in structure, size, and functionality to the target molecule.^27^ These polymeric materials have benefits such as chemical and physical stability, increased selectivity and sensitivity, simplicity in synthesis, reusability, and low-cost manufacturing.^28^

Several synthetic routes have been devised in recent years to synthesize them, and emphasis has lately shifted to their feasible applications in bioscience. Besides a wide range of interesting applications, growth factors were imprinted in order to improve the selectivity of a drug delivery system or easily entrap them, allowing them to function as an inhibitory mechanism.^28,29^

Combining imprinted polymers with MNPs offers potential for biological applications. MNPs have fascinating features, such as being used as contrast materials in magnetic resonance imaging and utilizing an external magnetic field to raise local temperature, resulting in the breakage of hydrogen bonds between molecules and the polymer.^30^ Furthermore, MNPs not only improve the mechanical and chemical stability of MIPs in the body but they also introduce unique magnetic separation.^30,31^

The long-term biocompatibility of MIPs is unclear. Based on our current knowledge, only a few articles address the toxicity of imprinted polymers, but none document the influence of magnetic MIPs on cell survival or destruction in a bioenvironment. However, further study is needed to assess their clinical safety and biocompatibility in the context of their medicinal uses.^32,33^ Oral drug delivery, the most convenient route for administration, has challenges such as poor absorption, instability of drugs in acidic/basic digestive system environments, and unwanted degradation. pH-Responsive systems solve these challenges by providing targeted and controlled drug release according to the pH variations throughout the gastrointestinal region.^34,35^

The current paper aims to introduce and optimize an inexpensive, efficient, simple, and rapid method to prevent poisoning and side effects of STL injection. For this purpose, pH-responsive MMIPs, MIPs, and MIP_F_ were synthesized through free radical polymerization to selectively absorb STL molecules in the body. Additionally, BMG as an antidote was loaded on nanocarriers to improve the effectiveness. The smart uptake and controlled release of STL by MMIPs indicate the most effective nanocarrier compared to other forms of synthesized polymers.

## 2. Experimental

### 2.1 Materials

Iron(iii) chloride (FeCl_3_·6H_2_O) (Samchun, Seoul, Republic of Korea), iron(ii) chloride (FeCl_2_·4H_2_O), tetraethyl orthosilicate (TEOS), methacrylic acid (MAA), ethylene glycol dimethacrylate (EGDMA), 3-trimethoxysilyl-propyl methacrylate (TMSPMA), and 2,2′-azobisisobutyronitrile (AIBN) were supplied by Sigma-Aldrich (Munich, Germany). Disodium hydrogen phosphate, sodium dihydrogen phosphate, potassium chloride, and solvents such as absolute methanol, ethanol, hydrochloric acid, and ammonia (25%) were purchased from Merck Co. Water was purified with a Milli-Q system, whose conductivity was at a constant 18.2 MΩ.

The monomer and initiator were purified from some inhibitors that were added by companies to prevent polymerization before use. AIBN was recrystallized using methanol (1 : 5) and MAA was distilled under reduced pressure in the presence of CuCl_2_.

### 2.2 Characterization apparatus

#### 2.2.1 Fourier transform infrared spectrometry (FTIR)

The structure of the produced nano-biocomposites was investigated using an FTIR spectrometer (Bruker Vector-22). Sample's wavenumber (cm^−1^) *vs.* transmittance percent was determined using a KBr pellet approach in the 400 to 4000 cm^−1^ range.

#### 2.2.2 X-ray diffraction (XRD)

Powder XRD diffractograms of produced nanocomposites were acquired from dried samples using a Bruker AXS-D8 Advance diffractometer. The results were employed with CuKα radiation (*λ* = 1.542 Å) over the Bragg angle of 2*θ* = 2–70°.

#### 2.2.3 Thermogravimetric analysis (TGA)

To study thermal stability of prepared nanocomposites, a TGA Q500 analyzer was used. Weighed nanomaterials were evaluated in a N_2_ (inert) environment from 47 to 800 °C at a heating rate of 10 °C min^−1^.

#### 2.2.4 Ultraviolet-visible spectrophotometry (UV-vis)

UV-vis spectra were obtained using a Philips PU 8620 ultraviolet spectrophotometer. All samples were placed in a 1 cm quartz cell and their absorbance was measured at the absorption maximum (*λ*_max_).

#### 2.2.5 Scanning electron microscopy (SEM)

The surface morphology of created products and elemental composition were determined by SEM. The specimens were covered with a thin layer of gold through sputtering to make them conductive for examination at 30 kV using a MIRA3 FESEM microscope, which is equipped with energy-dispersive X-ray spectroscopy (EDX).

#### 2.2.6 Dynamic light scattering (DLS)

In order to study the hydrodynamic size distribution, stability, and the polydispersity index (PDI) of nanocarriers under physiological conditions, they were evaluated using a DLS (Microtrac, Nanotrac Wave) in PBS at 25 °C.

### 2.3 Preparation of iron oxide nanoparticles (Fe_3_O_4_ NPs)

The magnetic nanoparticles of Fe_3_O_4_ were prepared by the coprecipitation method using ferric (Fe^3+^) and ferrous (Fe^2+^) chloride.^36,37^ In brief, 20 mmol of ferric chloride (FeCl_3_·6H_2_O) and 10 mmol of ferrous chloride (FeCl_2_·4H_2_O) were dissolved separately in 100 mL of deionized water (DW) and mixed into a two-neck round-bottom flask. Then the solution was purged under a N_2_ atmosphere for 30 min to be deoxygenated and stirred for one hour at room temperature (RT = 25 °C). The mixture was stirred vigorously (750 rpm) and heated to 80 °C. 40 mL of ammonia solution (NH_4_OH 25%) was added dropwise under constant stirring for one hour to change the color of the solution to black. The magnetic nanoparticles were precipitated using a strong magnet, washed with DW and ethanol three times to remove unreacted reagents, and dried using a vacuum desiccator.

### 2.4 Modification of Fe_3_O_4_ NPs with silyl groups (Fe_3_O_4_@SiO_2_)

This modification was performed by a modified Stöber method.^38^ Fe_3_O_4_ NPs (0.4 g) were dispersed in an ethanol–DW mixture (8 : 2) for two hours. 2 mL of NH_4_OH 25% and 4 mL of TEOS were added under sonication, transferred to a 40 °C hotplate, and stirred at 800 rpm for another 24 h. The products were washed with DW and ethanol several times, collected using an external magnet, and dried in a vacuum desiccator.

### 2.5 Vinylation of modified Fe_3_O_4_ NPs (Fe_3_O_4_@SiO_2_@TMSPMA) (MNPs)

The silica-coated magnetic nanoparticles (0.5 g in 100 mL ethanol : DW) were dispersed in an ultrasonic bath for one hour. After the addition of 2 mL 25% ammonia solution and 4 mL of TMSPMA dropwise, the mixture was vigorously stirred at 1000 rpm and heated at 60 °C for 24 h. The obtained materials were washed with DW and ethanol to eliminate any unreacted components following magnetic decantation. The materials were finally dried under vacuum and stored at 4 °C.

### 2.6 Preparation of magnetic MIP (MMIP) and non-magnetic MIP (MIP) powder

The template–monomer combination was prepared by dissolving STL (37.8 μmol) and MAA (0.1891 mmol) in 4 mL of dry THF. Then MNPs (40 mg) as the core, EGDMA (0.7564 mmol) as a cross-linker, and AIBN (3.1 mg) as the initiator were added and sonicated for another 10 minutes. After being purged with argon gas for 15 min, the mixture was sealed, gently agitated (250 rpm), and heated to 60 °C in a water bath for 48 hours. The resultant hard polymer was ground into a soft hemogenic powder and rinsed with water to eliminate excess and unreacted chemicals. Then it was centrifuged at 6000 rpm and dried at 40 °C. To remove the template, a “Soxhlet apparatus” was used for one week with 200 mL of methanol. For comparison, NIPs and MNIPs were synthesized using the same method but without the insertion of a template. In addition, MIPs without magnetic cores (MNPs) were created using the same process.

### 2.7 Preparation of MIP fiber (MIP_F_)

The molecularly imprinted fibers were manufactured using the previously disclosed method.^39^ 18.9 mmol STL and 0.945 mmol MAA were dissolved in THF for 10 min. Then, 3.782 mmol of EGDMA and 0.015 g of AIBN were added and placed in an ultrasonic bath for 10 min. The pre-polymer solution was degassed with an argon stream for 15 min before being poured into glass capillary tubes with internal dimensions of 4 cm × 0.53 mm. After sealing the capillary tubes, they were polymerized in a water bath at 60 °C for 48 h. The resulting polymeric monolith was removed from the mold and washed in water for 8 h. To remove the template, the fibers were thoroughly washed with methanol using a Soxhlet apparatus for one week before drying at 40 °C. In the absence of a template, the NIP fibers were synthesized using the same process.

### 2.8 *In vitro* loading study

By *in vitro* loading tests, the binding characteristics and selectivity of all produced MIP- and NIP- based nanocarriers can be recognized. For this aim, 5 mg of MIPs and NIPs were incubated separately in 5 mL of 20 ppm STL and BMG solutions at room temperature (25 °C) for 24 h. The amount of unbound STL in the supernatant was evaluated using a UV-vis spectrophotometer, and the quantity of adsorbed drugs on each MIP and NIP was calculated using a calibration curve. The collected all types of MIPs and NIPs were subsequently washed with DW to eliminate the physically adsorbed drugs and then dried under vacuum for 48 h.

### 2.9 *In vitro* release study

To investigate the release of STL and BMG, two different buffer solutions with pH values of 1 and 7.4 were used to stimulate the environments of the stomach and intestine, respectively. For this purpose, an adequate number of loaded MIPs and NIPs were deposited separately in 4 mL buffer solution in a dialysis bag (MWCO 12 000 Da, Sigma-Aldrich) and submerged in a 15 mL buffer solution at 37 °C for 78 h.^40^ At proper times, 3 mL of solution was removed and replaced with an equivalent amount of new buffer solution. The quantity of STL and BMG in the collected release media was quantified with a UV-vis spectrometer at wavelengths of 290 and 211 nm, respectively. It is important to note that MIPs and NIPs are researched concurrently and under identical conditions. The cumulative release of drugs was computed using the following equation:
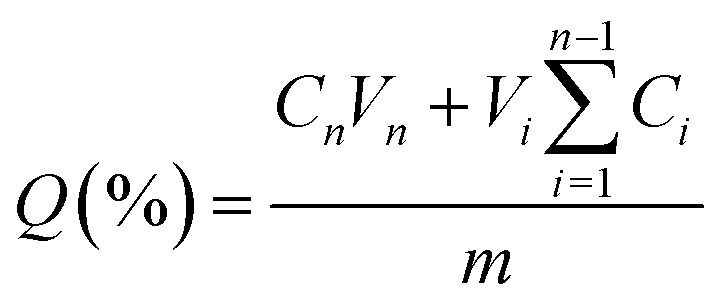
where *Q* is the proportion of total release and *C*_*n*_ and *V*_*n*_ indicate the concentration and volume of the release media. *C*_*i*_ and *V*_*i*_ are the drug concentration and volume of each sample, whereas *m* is the mass of the drug.

### 2.10 Statistical analysis

The obtained data were achieved by at least triplicate examinations and analyzed using analysis of variance (ANOVA) and the Student's *t*-test to find significant differences between groups. The difference was determined to be statistically significant when *P* values were less than 0.05. Data were presented as a mean ± SD.

## 3. Results and discussion

Fe_3_O_4_ NPs were produced as a core using the co-precipitation process in a neutral environment. Silyl groups modified the surface of magnetic iron nanoparticles, increasing the thermal stability, acid resistance, phase stability, and biocompatibility for biological and tissue engineering applications.

**Scheme 1:**
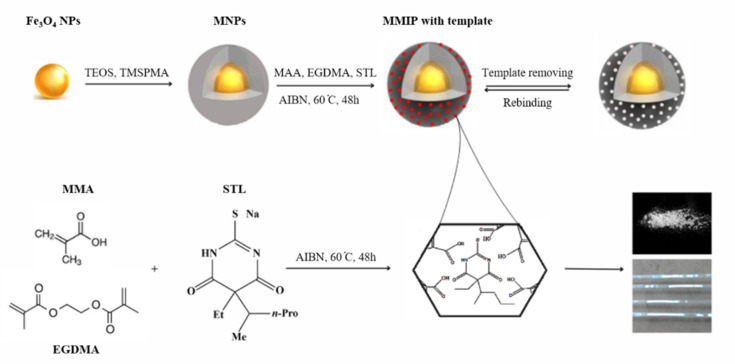
An illustration of MNPs and MMIP synthesis.

All kinds of MIPs were synthesized using the non-covalent approach, in which MIPs and NIPs were created concurrently and under the same circumstances. In radical polymerization, MAA served as the monomer, EGDMA as the crosslinker, AIBN as the initiator, STL as the template, and THF as the solvent. Scheme 1 shows an overview of the reactions that occur during the synthesis process.

### 3.1 FTIR

**Fig. 1:**
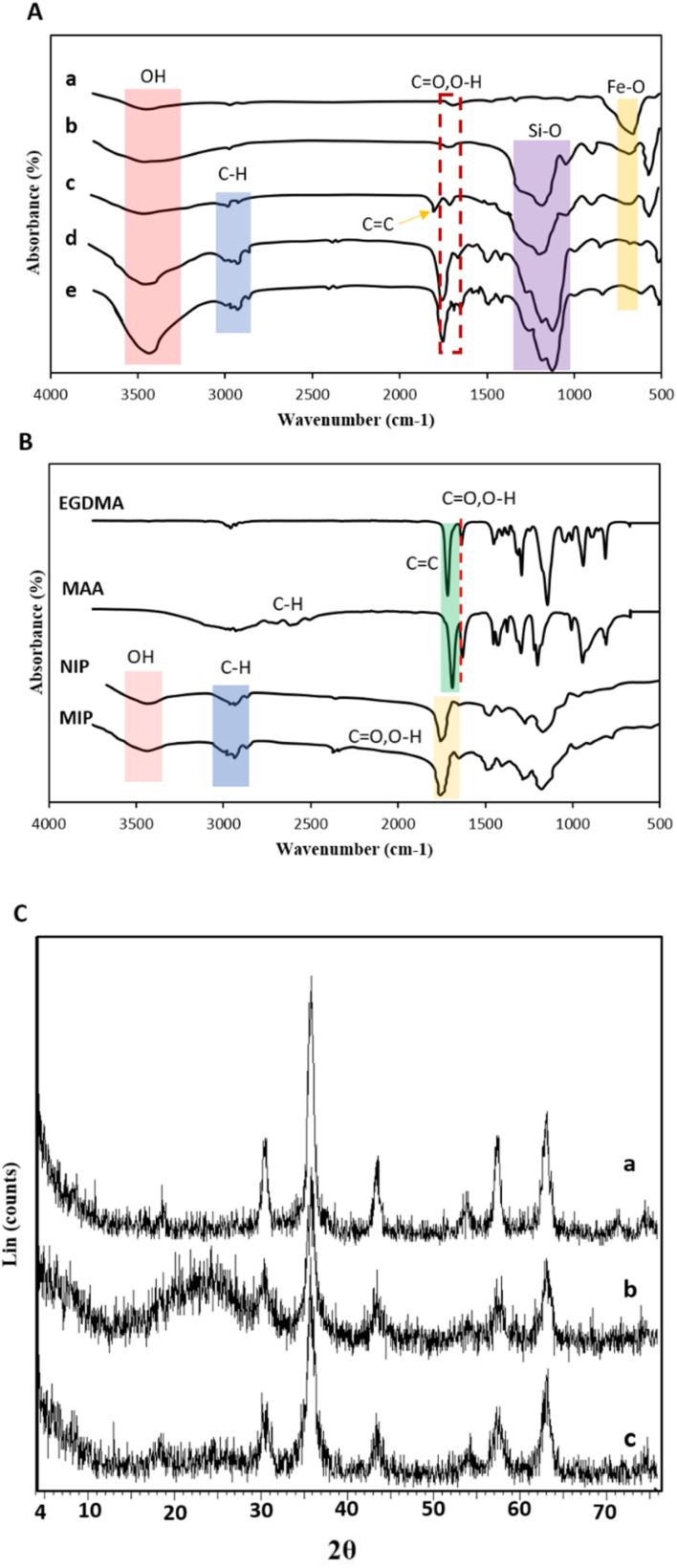
(A) FTIR spectra of (a) Fe_3_O_4_, (b) Fe_3_O_4_@SiO_2_, (c) Fe_3_O_4_@SiO_2_@TMSPMA, (d) MNIPs and (e) MMIPs; (B) FTIR spectra of the monomer, crosslinker, MIPs and NIPs; (C) XRD patterns of (a) Fe_3_O_4_, (b) Fe_3_O_4_@SiO_2_, and (c) Fe_3_O_4_@SiO_2_@TMSPMA.

The chemical structures and functional groups of synthesized nanomaterials were studied using FTIR analysis. As shown in Fig. 1Aa, the vibrational band of Fe–O–Fe and stretching and bending vibrations of –OH groups emerged at 565 cm^−1^, 3420 cm^−1^, and 1621 cm^−1^ on Fe_3_O_4_NPs, suggesting the existence of superior hydroxyl groups on the surface, ideal for silylation processes.^41^ After reacting with TEOS (Fig. 1Ab), the peak of Fe–O–Fe bonds reduced.^42^ Two additional sharp absorption peaks at 1092 and 801 cm^−1^ showed the symmetric and asymmetric stretching vibration of Si–O bonds, while the peak at 584 cm^−1^ suggested the Si–O–Fe bond.^43^Fig. 1Ac shows new peaks at 2853 cm^−1^, 2924 cm^−1^, 1637 cm^−1^, and 1718 cm^−1^, which correspond to the stretching vibrations of aliphatic C–H, C=O, and C=C, respectively. MNIPs and MMIPs in Fig. 1Ad and e showed all of the Fe_3_O_4_@SiO_2_@TMSPMA bonds, indicating that the polymers were effectively synthesized on the surface of Fe_3_O_4_ nanoparticles. According to Fig. 1B, the presence of a broad peak at 3420 cm^−1^ indicated the acidic –OH group; the peaks at 2925 cm^−1^ and 2855 cm^−1^ are related to aliphatic –CH; the sharp and strong peak at 1734 cm^−1^ is related to the C=O groups of the EGDMA crosslinker; and the peaks at 1157 cm^−1^ and 1635 cm^−1^ indicated the stretching band of the C–O and O–H groups.^44^ The C=C bonds of MAA and EGDMA at around 1689 and 1718 cm^−1^, respectively, disappeared in polymer spectra, showing complete polymerization. Finally, according to Fig. S2,† the STL peaks (C–N at around 1540 cm^−1^ and N–H stretching bond at 3249 cm^−1^) cannot be seen in spectra of washed MIPs.

### 3.2 XRD

The crystal structure of the core of nanocomposites (Fe_3_O_4_, Fe_3_O_4_@SiO_2_, and Fe_3_O_4_@SiO_2_@TMSPMA) was investigated using XRD. Fig. 1D shows diffraction peaks of Fe_3_O_4_NPs in the 2*θ* region (30.4°, 35.84°, 43.68°, 53.92°, 57.32°, and 63.28°) with crystalline planes of *d*220, *d*311, *d*400, *d*422, *d*511, and *d*440, respectively (JCPDS no. 19-0629), indicating the magnetite phase.^45,46^ Modifying Fe_3_O_4_NPs with TEOS reduced the strength of these peaks and revealed a wide peak at 2*θ* = 24°, suggesting the creation of amorphous SiO_2_ groups on the surface of Fe_3_O_4_NPs with no change in the crystalline structure.^31,47^ The characteristic peaks of Fe_3_O_4_NPs broadened partially and decreased in intensity which indicates the shell of TMSPMA on Fe_3_O_4_@SiO_2_.^47,48^ On the other hand, the breadth of the diffraction signals remains constant, suggesting a hemogenic dispersion with no aggregates in the coating layers of Fe_3_O_4_.

### 3.3 TGA

**Fig. 2:**
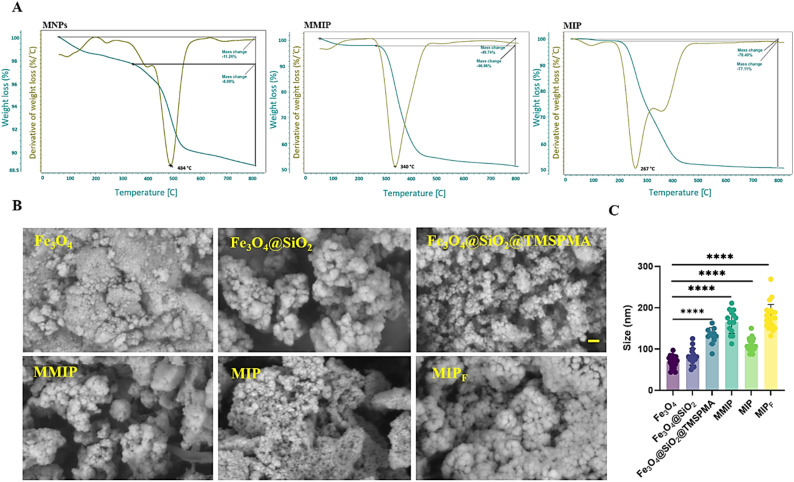
(A) TGA thermograms of MNPs, MMIPs, MIPs. (B) SEM images; the scale bar is 300 nm. (C) Size of nanocomposites. Data were used as mean ± SD, and *P* values were ****p < 0.0001 for all carriers by one-way ANOVA.

In order to investigate the impact of the magnetic core on thermal stability of MIPs, Fe_3_O_4_@SiO_2_@TMSPMA (MNPs), MMIPs, and MIPs were used here. According to Fig. 2A, the weight loss of pure MNPs of approximately 2.37% at 80–100 °C relates to moisture removal. The weight loss at 484 °C was caused by the loss of the silica coupling agent grafted onto the Fe_3_O_4_NPs.^49^ The MMIP thermogram showed a weight loss of roughly 2.8% between 80 and 100 °C, which was due to water evaporation. The weight loss at 340 °C (46.96%) indicated the breakdown of the grafted polymer on the shell of MNPs.^50^ The minimal weight loss at 80–120 °C in the MIP thermogram is caused by moisture elimination. The MIP decomposed at 267.5 °C, showing that it was less stable compared to grafted magnetic nanoparticles. TGA data show that thermal degradation of all nanocomposites (MNPs, MMIPs, and MIPs) begins at around 267.5 °C, indicating that they are absolutely stable at human body temperature.^43^

### 3.4 SEM and EDX

The surface morphology and particle size of synthesized Fe_3_O_4_, as well as MMIPs, MIPs, and MIP_F_, were investigated using SEM. Fig. 2B shows a homogeneous and nearly spherical structure formed by Fe_3_O_4_, with an average particle size of around 69.17 ± 13.7 nm.^51^ Following surface modification of Fe_3_O_4_NPs using TEOS and TMSPMA, a thin layer of silyl and acrylate functional groups was formed around monodispersed magnetic Fe_3_O_4_NPs, which did not affect their spherical shape but increased the agglomeration and diameter of particles.^47^ Various alterations occurred after the development of a polymer layer on the magnetic nanoparticle core with an enhancement in diameter (Fig. 2C).^52^ According to Fig. 2B, MIP and MIP_F_ structures exhibit sponge-like characteristics, indicating the lack of TPL medication when washed with Soxhlet equipment. These findings demonstrated the effective synthesis of MNPs and imprinting. Furthermore, the picture of the surface of MMIPs, MIPs, and MIP_F_ following drug loading demonstrates drug deposition on the polymers' surfaces.^51^ Fig. S3† displays the outcomes of the EDX study for samples, which support the SEM results. Table 1 summarizes the atomic percent of the synthesized nanocomposites and polymers.

**Table 1:** Atomic percents of the synthesized nanocomposites, polymers, and STL loaded on polymers

Component	Fe	O	Si	C	N	S
Fe_3_O_4_	36.1	63.9	—	—	—	—
Fe_3_O_4_@SiO_2_	5.9	71.3	22.9	—	—	—
MNPs	8.1	48.4	5.1	38.5	—	—
MMIPs	1.0	33.3	2.6	63.1	—	—
MIPs	—	22.6	—	77.4	—	—
MIP_F_	—	24.4	—	75.6	—	—
MMIP loaded	0.8	34.9	3.1	55.3	5.0	1.0
MIP loaded	—	22.7	—	70.5	5.7	1.1
MIP_F_ loaded	—	23.3	—	70.6	5.1	1.0

### 3.5 DLS

Stability of nanocarriers in a physiological environment is critical for bioapplications. In this regard, 1 mg of MMIPs and MIPs were separately dispersed in 5 mL PBS for 15 min. The average diameter and PDI of monodispersed MMIP and MIP particles were obtained as 152 nm, 0.192 and 107 nm, 0.131 respectively, on day 0, which is approximately similar to the particle size measured by SEM in the dry state. Dispersed carriers were kept in an incubator at 37 °C for one week. The resulting size and PDI showed an increase of approximately 462 nm, 0.23 for MMIPs and 396 nm, 0.188 for MIPs, indicating swelling in PBS over time and delivering more STL in the SIF environment.^43^

### 3.6 *In vitro* drug loading and release

**Fig. 3:**
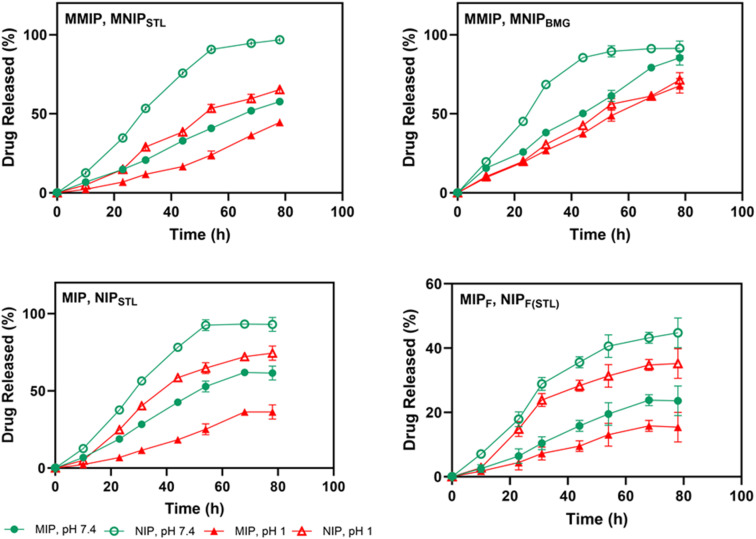
The *in vitro* release profiles of STL and BMG from prepared nanocarriers at pH 1 and pH 7.4 at 37 ± 1 °C for 78 h. Data were used as mean ± SD; the *P* values were ****p < 0.0001 for all carries by two-way ANOVA.

To investigate *in vitro* loading, STL was loaded onto all samples (MMIPs, MIPs, and MIP_F_, and their corresponding NIPs) overnight at room temperature (25 °C). MMIPs and MIP_F_ showed the highest and the lowest loading amounts of 19.6 and 11.37 μg mL^−1^ of nanocarriers, respectively. *In vitro* release behavior of BMG was investigated on MMIPs and MNIPs. STL and BMG have distinctive absorption peaks at 290 and 211 nm, respectively. Their calibration curves are displayed in Fig. S4.† UV-vis spectrophotometry was used to measure the drug loading concentration and encapsulation efficiency, which are presented in Table 2. SEM images in Fig. S5† and EDS data in Table 1 show the successful loading of STL molecules on produced nanocarriers. *In vitro* release tests were carried out for 78 hours at 37 ± 1 °C using a medium with varied pH values of SGF and SIF. According to the findings (Fig. 3), the little amount of STL released by the nanocarriers increased over time. Under the same conditions, NIP-based nanocarriers released more quickly than comparable MIPs and had a considerably shorter sustained-release time. This could be attributed to physical adsorption and unspecific binding places on the NIP surface. On the other hand, MIP-based nanocarriers demonstrated a gradual and regulated release due to their particular binding sites' strong STL sensitivity. In pH value investigation, STL exhibited more efficient release in the SIF environment (pH 7.4). The nanocomposites' carboxylic groups would get deprotonated, resulting in negatively charged carboxylate groups.^48,53^ STL, as a typically negative charge molecule, causes a significant release in basic media. MMIPs showed a remarkable smart release behavior around 57.7% of the loaded amount (11.31 μg mL^−1^) among MIP (8.03 μg mL^−1^) and MIP_F_ (2.68 μg mL^−1^) nanocarriers. MNPs may enhance the surface area of MMIPs, creating more binding sites for drugs. Furthermore, magnetic features allow for control of MMIP aggregation, potentially affecting drug loading and release kinetics.^54^ In addition, higher release of BMG from MMIPs (85.4%) against STL could indicate the unspecific bindings of BMG and selective properties of synthesized MMIPs to STL. It should be noted that the released amount in MMIPs is in accordance with the therapeutic range of STL.^55^

**Table 2:** Loading and encapsulation efficiencies of synthesized nanocarriers

	MMIPs	MNIPs	MIPs	NIPs	MIP_F_	NIP_F_	MMIP_BMG_	MNIP_BMG_
Encapsulation efficiency (%)	98.45 ± 0.332	96.88 ± 0.487	65.3 ± 1.94	61.72 ± 1.02	56.83 ± 1.45	43.64 ± 1.398	90.18 ± 1.68	81.54 ± 1.79
Loading efficiency (%)	393.8 ± 1.328	387.52 ± 1.948	261.2 ± 7.76	246.88 ± 4.08	227.32 ± 5.8	174.56 ± 5.592	360.72 ± 6.72	326.16 ± 7.16

## 4. Conclusions

In this study, we produced pH-sensitive magnetic and nonmagnetic molecularly imprinted polymers. To increase biocompatibility for MMIPs, Fe_3_O_4_ NPs were functionalized with silyl and acrylate groups. Thermally radical polymerization creates polymeric shells on the surface of MNP cores.

Among the several MIP types, MMIPs had the greatest drug loading capacity and the slowest release rate in the SIF environment. On the other hand, *in vitro* drug loading and release tests of BMG on MMIPs revealed STL-specific locations on polymers. Furthermore, due to their great magnetic responsiveness, MMIPs could be easily reused. This method shows potential in biomedical uses such as smart and controlled drug delivery of STL in SIF, both orally and *via* injection. Future research will involve crucial *in vitro* and *in vivo* biocompatibility tests to fully evaluate their potential for biomedical applications.

## Author contributions

A. Yari-Ilkhchi has designed and performed experiments, investigated the data, wrote the draft, and reviewed and edited the manuscript. A. A. Rafi has designed and performed experiments and analyzed the data. M. Mahkam has conceptualized, supervised, and oversaw the entire project. All authors reviewed and commented on the manuscript.

## Conflicts of interest

The authors have no financial conflict to declare.

## Supplementary Material

NA-007-D4NA00926F-s001
