# Supplementary material for: Design and development of pH-sensitive nanocarriers using molecularly imprinted polymers for the targeted delivery of sodium thiopental
Source: Nanoscale Adv. 2025 Feb 18;7(7):2039–46. doi: 10.1039/d4na00926f (PMC11833455; doi:10.1039/d4na00926f)
Supplement: NA-007-D4NA00926F-s001 [file NA-007-D4NA00926F-s001.pdf]

Supplementary Information (SI) for Nanoscale Advances.
This journal is © The Royal Society of Chemistry 2025

Supplementary Data

Synthesis of Various pH-responsive Molecularly Imprinted Nanocarriers for Selective Uptake and Targeted Delivery of Sodium Thiopental

Ayda Yari-Ilkhchi [a,b,c], Abdolrahim Abbaszad Rafi [d], Mehrdad Mahkam [a*],

[a] *Faculty of Science, Chemistry Department, Azarbaijan Shahid Madani University, Tabriz, Iran*
[b] *Faculty of Chemical and Metallurgical Engineering, Department of Chemical Engineering, Istanbul Technical University, Maslak, 34469 Istanbul, Turkiye.*
[c] *Faculty of Engineering and Natural Sciences, Sabanci University, 34956 Istanbul, Turkiye.*
[d] *FSCN Research Center, Mid Sweden University, Holmgatan 10, 851 70, Sundsvall, Sweden.*

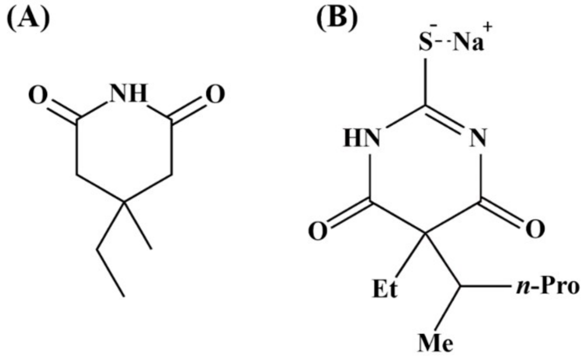

Fig. S1. The chemical structure of A: BMG and B: STL.

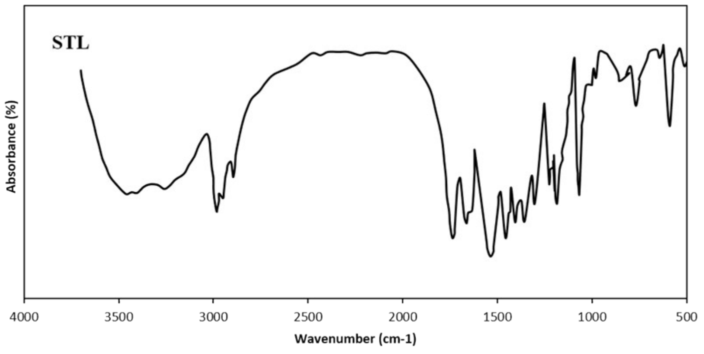

Fig. S2. The FTIR spectrum of STL

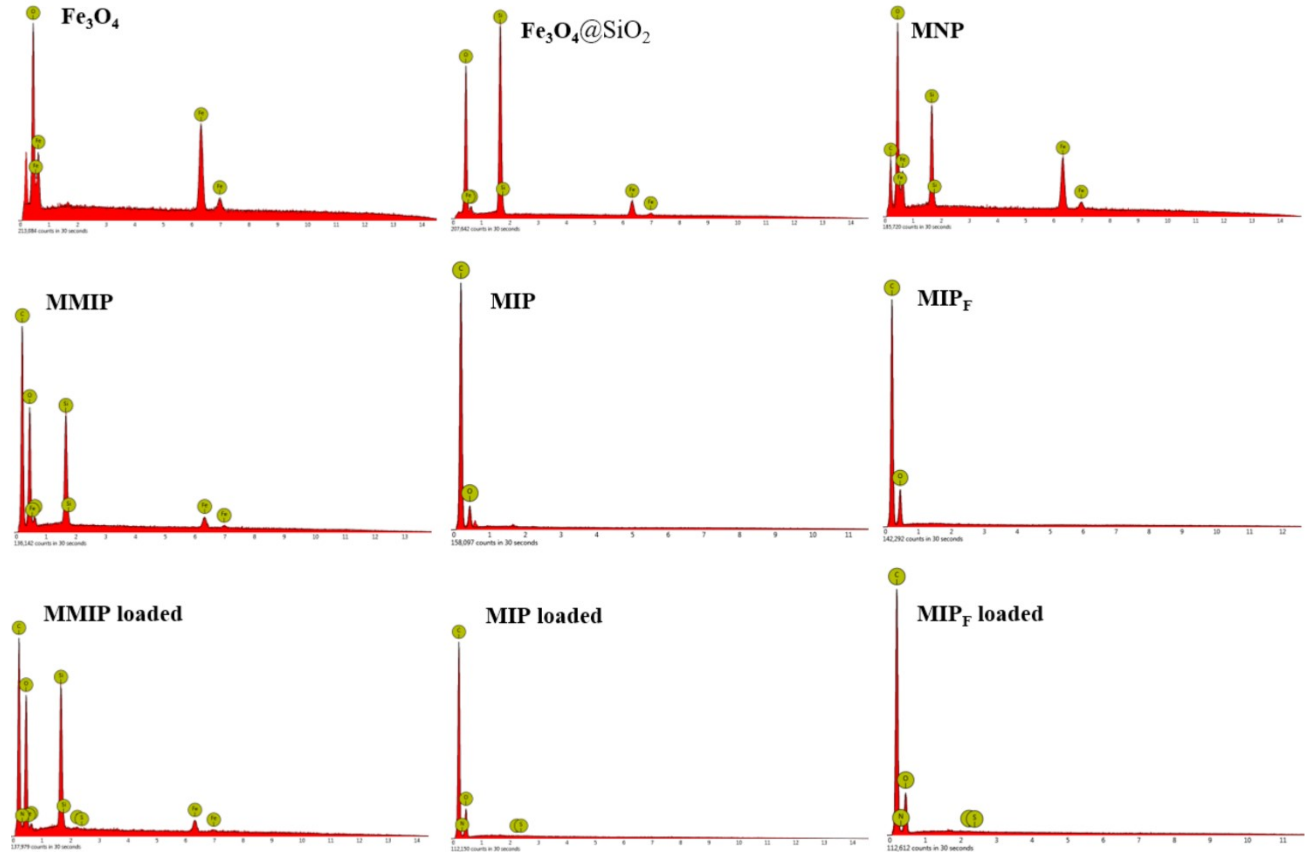

Fig.S3. EDX spectra of the prepared nanocomposites, polymers, and STL loaded on polymers.

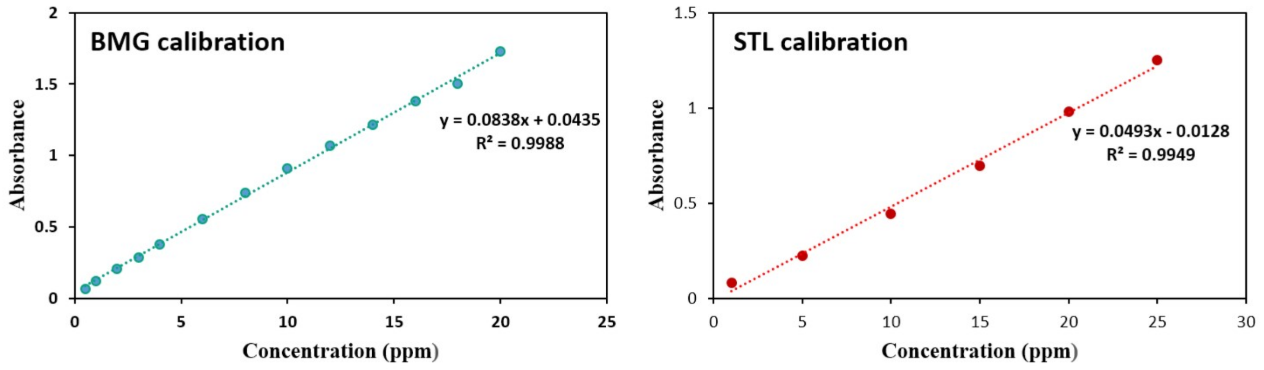

Fig.S4. The calibration curves of STL and BMG.

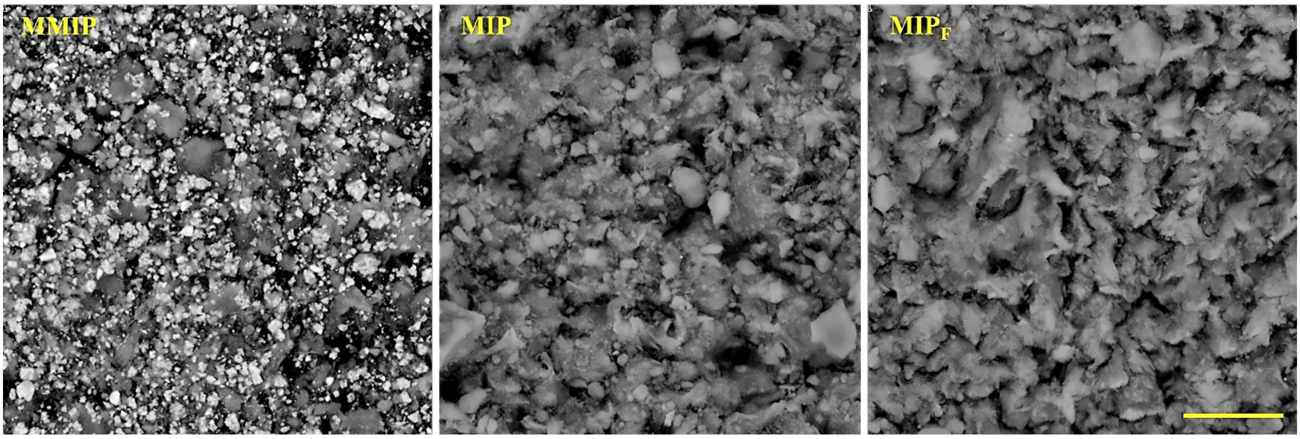

Fig.S5. SEM images of loaded STL on MMIP, MIP, and MIP_F nanocarriers. The scale bare is 20 μm.
